# Pomegranate-derived nanovesicles as therapeutic agents for acute pancreatitis in a murine model: anti-inflammatory, antioxidant, and endocrine-protective properties

**DOI:** 10.20517/evcna.2025.145

**Published:** 2026-03-10

**Authors:** Isabel Torres-Cuevas, Christian M. Sánchez-López, Antonio Marcilla, Salvador Pérez

**Affiliations:** ^1^Department of Physiology, Universitat de València, Valencia 46010, Spain.; ^2^Neonatal Research Group, Health Research Institute La Fe (IISLAFE), Valencia 46026, Spain.; ^3^Parasites and Health Group, Àrea de Parasitologia, Departament de Farmàcia i Tecnologia Farmacèutica i Parasitologia, Universitat de València, Burjassot 46100, Spain.; ^4^Joint Research Unit on Endocrinology, Nutrition and Clinical Dietetics, Health Research IIS La Fe-Universitat de València, Valencia 46026, Spain.; ^#^These authors contributed equally to this work.

**Keywords:** Pomegranate-derived nanovesicles, inflammation, oxidative stress, acute pancreatitis, endocrine dysfunction

## Abstract

**Aim:** Acute pancreatitis (AP) is an inflammatory disorder of the pancreas lacking specific therapy and frequently complicated by oxidative stress (OS) and long-term endocrine dysfunction, including pancreatogenic diabetes. Pomegranate-derived nanovesicles (*Pg*NVs) contain bioactive lipids, proteins, and metabolites responsible for many health benefits of pomegranate juice. This study evaluated whether prophylactic *Pg*NVs administration could mitigate pancreatic injury, OS, systemic inflammation, and subsequent endocrine impairment in a murine model of severe L-arginine-induced AP.

**Methods:** Male C57BL/6J mice were distributed to three groups: control, AP, and AP + *Pg*NVs. A single subcutaneous dose of *Pg*NVs (10 µg; ≈ 5 × 10^10^ particles) was administered 2 h before AP induction via intraperitoneal L-arginine. Animals were analyzed at 3, 7, 30, and 60 days post-induction. *Pg*NVs were isolated using differential centrifugation, tangential-flow filtration, and size-exclusion chromatography.

**Results:** *Pg*NVs pretreatment preserved pancreatic architecture, reduced edema, amylase activity, and interleukin 6 (IL-6) levels in both tissue and plasma by limiting nuclear factor-κB-p65 phosphorylation. *Pg*NVs restored redox balance by upregulating NQO1 [NAD(P)H quinone dehydrogenase 1], improving oxidized glutathione (GSSG)/reduced glutathione (GSH) and homocystine/homocysteine ratios, and maintaining PGC-1α (peroxisome proliferator-activated receptor gamma coactivator 1-alpha) expression. *Pg*NVs protected β-cell mass, enhanced insulin secretion, and normalized glucose tolerance after AP. Proteomic profiling revealed that *Pg*NVs changed extracellular vesicle composition, lowering pro-inflammatory markers [fibronectin, dipeptidyl peptidase-4 (DPP4)] and restoring anti-inflammatory ITIH4 (inter-alpha-trypsin inhibitor protein heavy chain 4).

**Conclusion:** *Pg*NVs exert anti-inflammatory and antioxidant protection in experimental AP and preserve long-term endocrine function, highlighting their potential as a natural nanotherapeutic strategy to prevent pancreatic injury and post-pancreatitis diabetes.

## INTRODUCTION

The pomegranate (*Punica granatum* L.) juice has been the focus of considerable research and application due to its valuable therapeutic properties, including wound-healing, anti-inflammatory, and antioxidant effects^[1]^. These beneficial properties are largely attributed to the high concentrations of phenolic compounds contained in the juice, such as tannins and anthocyanins^[2,3]^.

Plant-derived nanovesicles (PDNVs) have emerged as promising bioactive particles with clinical applications, driving an area of research experiencing exponential growth^[4]^. PDNVs are nano-sized structures (30-200 nm in diameter) with a lipid bilayer containing a diverse array of molecules, including proteins, nucleic acids (DNA and RNA), lipids, and metabolites. Due to their plant origin, PDNVs contain secondary metabolites not found in mammalian-derived extracellular vesicles (EVs), which confer distinct natural bioactivity to PDNVs. Additionally, PDNVs have demonstrated low toxicity, easy internalization by mammalian cells, and interesting anti-inflammatory and immunomodulatory properties. These attributes, combined with their wide availability, position PDNVs as natural alternatives for treating various pathologies^[5]^.

Our group first described the isolation of pomegranate-derived nanovesicles (*Pg*NVs), demonstrating their anti-inflammatory, antioxidant, and wound-healing properties *in vitro*, and characterizing their protein content^[6]^. Additionally, these functional properties are more potent than those observed in other constituents of the juice, highlighting *Pg*NVs as central to the bioactive properties of pomegranate juice^[6]^. These make *Pg*NVs a suitable potential treatment for inflammatory diseases, such as acute pancreatitis (AP).

AP refers to an inflammatory disorder of the pancreas, for which currently there is no specific treatment, often resulting in local and systemic complications^[7]^. Oxidative stress (OS) is a key factor in the etiopathogenesis of AP and in the development of its complications^[8]^; for this reason, antioxidant therapy using natural compounds has been widely employed^[9]^.

Recent observations indicate that among long-term complications, 40% of AP patients develop pancreatic endocrine insufficiency, which can evolve into a pre-diabetic state, or even to the development of *diabetes mellitus* (DM), requiring lifelong treatments or insulin^[10,11]^. This type of secondary complication of AP has been recognized clinically as a new entity called pancreatogenic diabetes or type 3c DM (T3cDM), differing from type 1 and type 2 DM in several important aspects^[12]^. It is noteworthy that T3cDM, although less common than type 2 DM, has surpassed type 1 DM in incidence, becoming the second cause of diabetes in adults^[12]^, representing between 5%-10% of hospitalized diabetic patients^[13]^. Furthermore, T3cDM has been associated with increased mortality risk and high likelihood of developing pancreatic cancer when compared to type 2 DM, mostly due to poorer glycemic control^[14]^. Consequently, prophylactic strategies are necessary for its prevention. Different *in vivo* studies have shown important hypoglycemic effects for pomegranate juice, as well as an improvement in insulin secretion^[15]^. In this context, *Pg*NVs, an important constituent of pomegranate juice, might be a good candidate in supplement formulations to prevent inflammatory disorders. In this study, we explore the preventive role of *Pg*NVs in a rodent model of AP, evaluating their functional properties *in vivo*.

## METHODS

### Animals and experimental model of severe AP

A total of 60 male C57BL/6J mice (The Jackson Laboratory, Barcelona, Spain) were maintained under standard environmental conditions with free access to food and water.

All experimental procedures were performed in accordance with Spanish regulations governing the use of animals for scientific purposes (RD 53/2013) and European Union legislation (Directive 2010/63/EU). The experimental protocols were reviewed and approved by the Ethics Committee for Animal Experimentation and Welfare of the University of Valencia, as well as by the regional authority (Conselleria de Agricultura, Ganadería y Pesca; approval code 2022/VSC/PEA 0285, type 3). In addition, the study was conducted in compliance with the Animal Research: Reporting of *In Vivo* Experiments (ARRIVE) guidelines.

Severe AP was induced in 8-week-old mice by 3 intraperitoneal (*i.p.*) L-arginine injections (Merck, Darmstadt, Germany) (3 g/kg body weight) at 1 h intervals^[16]^. The control group received physiological saline (0.9% NaCl).

**Figure 1:**
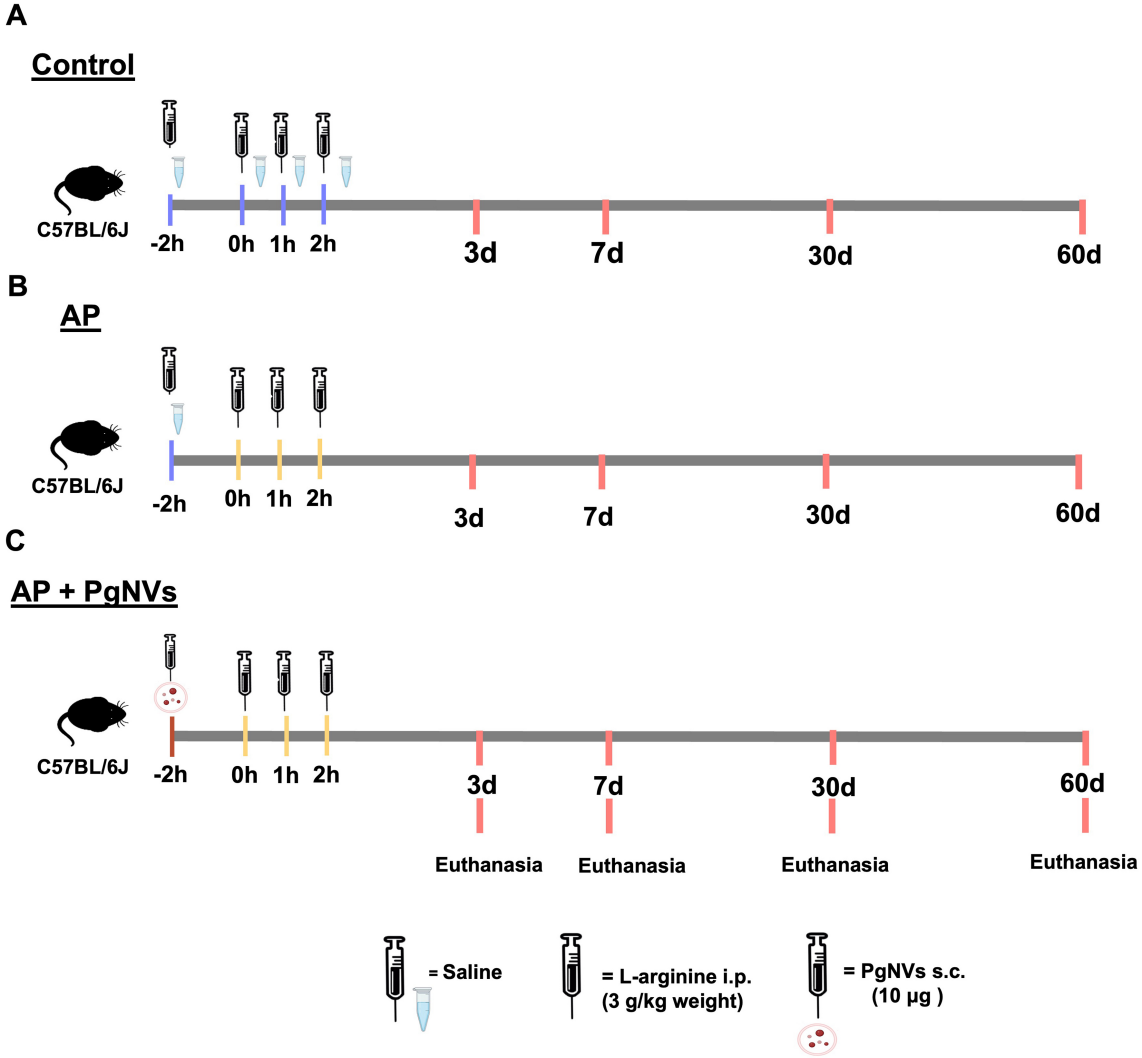
Experimental design: model of L-arginine-induced acute pancreatitis. Schematic representation showing distribution of the study groups: (A) Control group, injected with saline buffer (in blue); (B) Acute pancreatitis group, injected with L-arginine (in yellow); (C) Acute pancreatitis with *Pg*NVs treatment 2 h before L-arginine administration. AP: Acute pancreatitis; P*g*NVs: pomegranate-derived nanovesicles.

Mice were randomly assigned to three experimental groups: control (*n* = 20), AP (*n* = 20), and AP with *Pg*NVs (*n* = 20). *Pg*NVs were administered subcutaneously with a dose of 10 μg (corresponding to approximately 5 × 10^10^ particles) 2 h before the first L-arginine injection. Once AP was induced, the animals were euthanized under anesthesia with isoflurane 3%-5% at 3, 7, 30 and 60 days post-induction, with *n* = 5 in each experimental group for each time [Figure 1]. Different batches of animals were used at each experimental time point, and the measurements do not represent repeated measurements in the same subjects.

### Juice extraction and *Pg*NVs isolation

Juice extraction and isolation of *Pg*NVs were carried out according to our previously established protocol^[6]^. Briefly, pomegranate (*Punica granatum* L., var. “Mollar de Elche”) was purchased from a market in the Valencian Community, Spain. Prior to juice extraction, the fruits were thoroughly rinsed with distilled water. Juice was manually obtained using a hand-press juicer and subsequently filtered through a stainless-steel sieve (1 mm mesh) to remove solid residues.

To eliminate cellular debris and larger particles, the juice samples underwent a stepwise centrifugation process. Initially, the samples were spun at 700 × *g* for 10 min, followed by 3,000 × *g* for 15 min, and finally at 20,000 × *g* for 20 min at 4 °C to remove larger vesicles and contaminants. The subsequent supernatant (~200 mL) was concentrated using tangential flow filtration (TFF) with a Vivaflow^TM^ 50R membrane (Sartorius) connected to a Masterflex® L/S peristaltic pump (Cole-Parmer), reducing the volume to approximately 4-5 mL. Concentrated juice was divided into 1 mL aliquots and stored at -80 °C until further use.

*Pg*NVs were then isolated by size-exclusion chromatography. A 12 mL syringe (Sigma-Aldrich) was packed with 10 mL of Sepharose^TM^-CL2B resin (Sigma-Aldrich) and equilibrated with 0.22 µm filtered phosphate-buffered saline (PBS). One milliliter of the concentrated juice was loaded onto the column, and 20 fractions of 0.5 mL each were collected using PBS as the eluent. Fractions enriched in nanovesicles (NVs; fractions 6-10) were pooled and further concentrated using Amicon^TM^ Ultra-4 Centrifugal Filter units (Millipore) at 3,000 × *g* for 15 min at 4 °C. The purified *Pg*NVs were stored at 4 °C and used within two weeks of isolation. For each *Pg*NVs batch, particle concentration and protein content were quantified by nanoparticle tracking analysis (NTA) and bicinchoninic acid (BCA), respectively, ensuring a particle/protein ratio ≥ 5 × 10^9^ particles/µg protein, and typical morphology was assessed by transmission electron microscopy (TEM); batches failing these criteria were excluded from functional assays.

### Plasma collection and EVs isolation

Blood samples were extracted from animals 3 days post-AP induction with citrate as an anticoagulant and centrifuged at 1,000 × *g* for 15 min to obtain plasma. The obtained plasma was then centrifuged at 3,000 × *g* for 15 min, and subsequently at 20,000 × *g* for 20 min and 4 °C to eliminate larger EVs and impurities. Plasma EVs were isolated using a 1 ml syringe (Supelco) containing 1 mL of stacked Sepharose^TM^ CL-2B (Sigma-Aldrich) pre-washed with filtered PBS. A total of 150 µL of the resultant supernatant was introduced onto the column, and 12 fractions of 75 µL each were eluted via filtered PBS. Plasma EVs-enriched fractions (4-7) were then pooled, stored at 4 °C and used within a maximum of two weeks after isolation.

### *Pg*NVs and plasma EVs characterization

#### Protein quantification

The protein content of the *Pg*NVs samples was quantified with the Micro Bicinchoninic Acid (BCA^TM^) Protein Assay Kit (Thermo Fisher Scientific, Waltham, USA). For each measurement, 25 µL of the sample were combined with 25 µL of Milli-Q® water containing 0.05% Triton^TM^ X-100 and 0.01% sodium dodecyl sulfate (SDS) (Sigma-Aldrich). Absorbance was recorded at 595 nm using an iMark^TM^ Microplate Absorbance Reader (Bio-Rad, Hercules, CA, USA).

#### NTA

The NVs were characterized in terms of size distribution and particle concentration via NTA on a NanoSight LM10 instrument (Malvern Instruments Ltd.), following the approach reported by Sánchez-López *et al*. (2022)^[6]^. Imaging was performed with manual temperature monitoring. Prior to analysis, samples were diluted in 0.22 µm filtered PBS to achieve the manufacturer’s recommended particle concentration (20-120 particles per frame).

#### TEM

TEM analysis was conducted as previously described with slight modifications^[6]^. In brief, 6.5 µL of *Pg*NVs-enriched samples were fixed in 2% paraformaldehyde (PFA) for 30 min and placed onto carbon-coated grids for 15 min. Subsequently, samples were post-fixed with 1% glutaraldehyde for 5 min and rinsed with distilled water. Contrast staining was performed using a mixture of 1% uranyl acetate and 0.5% methyl cellulose. A HITACHI HT7800 electron microscope was used [Servicio Central Soporte e Investigación Experimental (SCSIE)].

### Cell culture conditions and NF-κB activation screening assay

The RAW 264.7 mouse macrophage cell line (ATCC®, ref. TIB-71) was used to assess the transcriptional changes induced by *Pg*NVs in the expression of inflammatory cytokines. To evaluate the changes in Nuclear Factor kappa-light-chain-enhancer of activated B cells (NF-κB) activation following treatment with mouse plasma EVs, we employed the RAW-Blue^TM^ cell line (InvivoGen, San Diego, CA), which is derived from RAW 264.7 cells. These cells contain a chromosomally integrated secreted embryonic alkaline phosphatase (SEAP) reporter system that responds to NF-κB transcriptional activation. Both cell types were maintained in Dulbecco’s Modified Eagle’s Medium (DMEM) supplemented with high glucose and glutamine (Gibco), 10% fetal bovine serum (FBS) (Gibco), 100 U/mL penicillin and 100 μg/mL streptomycin (both from Sigma-Aldrich). Cells were preserved in 75 cm^2^ flasks under an atmosphere of 5% CO_2_ and 100% humidity at 37 °C. NF-κB expression was assessed using the Quanti-Blue^TM^ colorimetric reagent (InvivoGen, San Diego, CA), which allows for monitoring SEAP production, following the manufacturer’s guidelines. Cells were seeded into 96-well plates at a concentration of 2 × 10^4^ cells/well, and cultured for 48 h at 37 °C, with 5% CO_2_ and 100% humidity. The macrophages were then treated with PBS, plasma EVs from control mice (Control EVs) (10 µg/mL), plasma EVs from mice with induced AP (AP EVs) (10 µg/mL), AP EVs and pretreated with *Pg*NVs (AP + *Pg*NVs EVs) (10 µg/mL), or 300 ng/mL of lipopolysaccharide (LPS) from *Escherichia coli* K12 (InvivoGen) as a pro-inflammatory control. After a 24-h incubation at 37 °C, with 5% CO_2_ and 100% humidity, 20 μL of cell culture supernatant was mixed with 180 μL of Quanti-Blue^TM^ reagent and incubated for 5 h at 37 °C. The optical density (OD) was measured at 655 nm using an iMark^TM^ microplate reader (Bio-Rad).

### Proteomic analyses

EVs isolated from plasma of mice at 3 days post AP induction were analyzed by liquid chromatography-tandem mass spectrometry (LC-MS/MS) as previously described^[6]^. Three replicate samples were processed using the automated sample preparation pipeline for low-input proteomics based on the single-pot solid-phase enhanced sample preparation (SP3) method^[17]^ performed at the SCSIE, Universitat de València (UV), Spain. Before processing, a 1:1 mixture of two types of carboxylate-modified magnetic particles (Thermo Scientific Sera-Mag SpeedBeads) was washed three times with 1 mL of water. The beads were placed on a MagneSphere magnetic rack (Promega), and the supernatant was removed after each wash. Afterward, the beads were resuspended in 500 μL of water and stored at 4 °C as a 20 μg/μL stock concentration.

For sample preparation, a volume equivalent to 35 µg of protein of control, AP or AP + *Pg*NVs EVs was dried and reconstituted with 30 μL of 50 mM ammonium bicarbonate (ABC). To each sample, 4 μL of beads were added, followed by acetonitrile (ACN) to reach a final concentration of 70%. The mixture was vortexed briefly, incubated for 20 min at room temperature, and then placed on a magnetic rack for 2 min, with the supernatant discarded. The beads were washed twice with 200 μL of 70% ethanol (EtOH) and once with 180 μL of ACN, each wash followed by a 30 s incubation on the magnetic rack and removal of the supernatant. The beads were dried for 2 min to ensure complete removal of ACN.

For elution, the beads were resuspended in 45 μL of 50 mM ABC buffer and treated overnight with 500 ng of trypsin (Promega) at 37 °C in a final volume of 50 μL. Digestion was halted by adding 60 μL of 10% trifluoroacetic acid (TFA) (Sigma-Aldrich), adjusting the final pH to 1. After centrifugation for 1 min to pellet the beads, the supernatant containing peptides was collected using a plate magnet. The peptide mixtures were concentrated by speed vacuum to a final volume of 10 μL.

The final peptide mixtures were analyzed by LC-MS/MS. Five microliters of the resulting mixture were loaded onto a trap column (3 μm C18-CL, 350 μm × 0.5 mm; Eksigent), desalted with 0.1% TFA at a flow rate of 5 μL/min for 5 min. Peptides were then separated on an analytical column (3 μm C18-CL 120 Å, 0.075 mm × 150 mm; Eksigent) equilibrated with 5% ACN and 0.1% formic acid (FA). Elution was performed using a linear gradient of 15%-40% buffer B in buffer A over 60 min (A: 0.1% FA; B: 5% ACN and 0.1% FA) at a flow rate of 300 nL/min. Peptides were analyzed using a nano-electrospray ionization (nanoESI) hybrid quadrupole-quadrupole-time-of-flight (QqTOF) mass spectrometer (6600plus TripleTOF, ABSciex). Ionization was achieved using an Optiflow < 1 μL Nano source with a spray voltage of 3.0 kV at 175 °C. Data were acquired in data-dependent acquisition mode, with MS1 survey scans from 350-1,400 m/z for 250 ms. MS2 scans were acquired at 100-1,500 m/z for 25 ms in high sensitivity mode. Protein identification was performed using the ProteinPilot v5.0 search engine and the Paragon algorithm^[18]^, searching the Swiss-Prot database. For label-free quantitative proteomics, peptide areas were normalized by total area, followed by differential analysis (DA) and *t*-tests using MarkerView 1.3 (Sciex). Missing Label-Free Quantification (LFQ) values were replaced with a limit of detection (LOD) value of 18.696, corresponding to the lowest detected LFQ value in the dataset (protein accession Q62261). This substitution allowed all proteins to be included in downstream differential expression analyses.

### Oral glucose tolerance test

Oral glucose test tolerance (OGTT) was performed based on the work of Andrikopoulos *et al*.^[19]^. Briefly, after 6 h of fasting, mice received a glucose dose of 2 g/kg, and blood samples were collected from the tail vein of mice in each group, and measured at 0, 30, 60, 90, and 120 min by a Contour Next Glucometer (Ascensia Diabetes Care, Barcelona, Spain). OGTT was performed at 7, 30, and 60 days after AP induction.

The area under the curve (AUC) was calculated by trapezoidal approximation of blood glucose levels^[20]^.

### Plasma determinations

Blood samples were collected in citrate as an anticoagulant and centrifuged at 1,000 × g for 15 min to obtain plasma. Amylase activity was determined in plasma using the AMYLASE-LQ kit (Spinreact, Spain) following the manufacturer’s instructions. A mouse interleukin (IL)-6 Quantikine® enzyme-linked immunosorbent assay (ELISA) kit (R&D Systems, Minneapolis, USA) was used to measure plasma interleukin 6 (IL-6) levels, with a monoclonal antibody specific for mouse IL-6 pre-coated onto a microplate as indicated in the kit instructions. Insulin plasma levels were determined using the RayBio® Mouse Insulin ELISA Kit (RayBiotech, Georgia, USA).

### RNA extraction and RT-qPCR analysis

Total RNA was isolated using TRIzol^TM^ reagent (Merck, Darmstadt, Germany) following the manufacturer’s instructions. The RNA concentration was measured in a NanoDrop Lite spectrophotometer (Thermo Scientific, Waltham, MA, USA), and purity was determined by OD 260/280 ratio. RNA was reverse transcribed to complementary DNA (cDNA) with the PrimeScript^TM^ RT Reagent Kit (Perfect Real Time) (Takara Bio Inc., Kusatsu, Japan) following the manufacturer’s instructions. Gene expression measured as RNA levels was performed in a thermal cycler [I-Cycler + IQ Multicolor Real-Time Optical Cycle Reader (OCR) Detection System, Biorad, Hercules, CA, USA] using the SYBR® Green Polymerase Chain Reaction (PCR) Master Mix (Takara Bio Inc., Kusatsu, Japan) or TaqMan PCR Master Mix (Applied Biosystems, Life Technologies Corporation, Carlsbad, CA, USA). Specific Taqman probes (all from Applied Biosystems, Life Technologies Corporation, Carlsbad, CA, USA) were used for cytokine analysis. The oligonucleotides for reverse transcription real-time quantitative PCR (RT-qPCR) are described in Table 1.

**Table 1:** The oligonucleotides used for RT-qPCR

**Target gene**	**TaqMan**
*Il1b*	Mm00434228_m1
*Il6*	Mm00446190_m1
*Tnfa*	Mm00443258_m1
*Tbp*	Mm01277042_m1
**Target gene**	**Forward/reverse oligonucleotide**
*Nqo1*	5’-TTCTCTGGCCGATTCAGAG-3’ 5’-GGCTGCTTGGAGCAAAATA-3’
*Nfe2l2*	5’- CCAGCAGGACATGGATTTG-3’ 5’- TGGAGTTGCTCTTGTCTTTCC-3’
*Ppargc1a*	5’-TTAAAGTTCATGGGGCAAGC-3’ 5’- TAGGAATGGCTGAAGGGATG-3’
*Tbp*	5’-CAGCCTTCCACCTTATGCTC-3’ 5’-CCGTAAGGCATCATTGGACT-3’

*Il1b*: *Interleukin 1 beta*; *Il6*: *interleukin 6*; *Tnfa*: *Tumor necrosis factor alpha*; *Tbp*: *TATA-box binding protein*; *Nqo1*: *NAD(P)H quinone dehydrogenase 1*; *Nfe2l2*: *nuclear factor (erythroid-derived 2)-like 2* (*Nrf2*); *Ppargc1a*: *Peroxisome proliferator-activated receptor gamma coactivator 1-alpha* (PGC-1α).

For amplification, an initial denaturation at 95 °C for 30 s, primer annealing at 60-62 °C for 30 s - with TaqMan probes at 60 °C; NQO1 [NAD(P)H quinone dehydrogenase 1] oligonucleotides at 60 °C; and *Ppargc1a* (*Peroxisome proliferator-activated receptor gamma coactivator 1-alpha*) and *Tbp* (*TATA-box binding protein*) oligonucleotides at 62 °C - and an extension at 72 °C for 1 min. These steps were repeated for 40 cycles.

The results were normalized using the TATA binding protein (*Tbp*) as housekeeping gene. The threshold cycle (CT) was determined, and the relative gene expression was expressed as follows: fold change = 2^-Δ(ΔCT), where ΔCT = CT target - CT housekeeping, and Δ(ΔCT) = ΔCT treated - ΔCT control.

### Western blot analysis

Pancreatic tissue samples were stored at -80 °C until homogenization, which was performed on ice using a Politron Generator FSH-G 5/085 (Thermo Fisher Scientific, Waltham, MA, USA) in lysis buffer prepared at 100 mg/mL. The lysis buffer contained 20 mM Tris-HCl (pH 7.5), 1 mM Ethylenediaminetetraacetic acid (EDTA), 150 mM NaCl, 0.1% SDS, 1% Igepal®, 30 mM sodium pyrophosphate, 50 mM sodium fluoride, and 1 mM sodium orthovanadate, supplemented with a protease inhibitor cocktail (Merck, Darmstadt, Germany) at 4 μL/mL. Homogenates were centrifuged at 15,000 rpm for 15 min at 4 °C. Protein concentration was determined using the bicinchoninic acid (BCA^TM^) protein assay (Thermo Fisher Scientific, Waltham, MA, USA).

The following primary antibodies were used for immunoblotting: anti-β-tubulin (1:1,000 ab6046, Abcam, Cambridge, UK), anti-NQO1 (1:1,000 ab80588, Abcam, Cambridge, UK), anti-phospho-p65 (Ser536) (1:100, #3033, Cell Signaling Technology, MA, USA), anti-Glyceraldehyde-3-phosphate dehydrogenase (GAPDH) (14C10) (1:1,000 #2118, Cell Signaling Technology, MA, USA), anti-vinculin (1:1,000 #4650, Cell Signaling Technology, MA, USA), and anti-peroxisome proliferator-activated receptor gamma coactivator 1-alpha (PGC-1α; 1:500, #4259, Cell Signaling Technology, MA, USA), all raised in rabbit. A rabbit secondary antibody (1:5,000, 7074, Cell Signaling Technology, MA, USA) was applied. Signals were detected using a chemiluminescence substrate [Enhanced Chemiluminescence (ECL); Fisher Scientific, Madrid, Spain] and captured with a ChemiDoc XRS Imaging System (Bio-Rad, Richmond, CA, USA). Band intensity was quantified using Image Lab software, version 2.0.1 (Bio-Rad, Richmond, CA, USA).

### Redox pairs by UPLC-MS/MS analysis

Redox pairs, specifically oxidized glutathione (GSSG)/reduced glutathione (GSH) and homocystine (HCyss)/homocysteine (HCys), were measured in pancreas samples that had been frozen and subsequently homogenized in PBS containing 10 mM N-ethylmaleimide. Following homogenization, perchloric acid was added to reach a final concentration of 4%, and the mixtures were centrifuged at 15,000 × *g* for 15 min at 4 °C. The levels of these analytes in the resulting supernatants were quantified using ultra-performance liquid chromatography coupled with tandem mass spectrometry (UPLC-MS/MS). The procedure was carried out according to the method described by Escobar *et al.*^[21]^

### Histological and immunohistochemical analysis

Tissue samples from the pancreas were extracted and fixed by immersion in 4% PFA. The pancreas was then dehydrated in EtOH baths with increasing concentrations and embedded in paraffin. These samples were cut to 5 µm thickness with a microtome (Leica, RM2125RT, Wetzlar, Germany).

Pancreas sections were stained with hematoxylin and eosin (HE) (Sigma-Aldrich, St. Louis, MO, USA).

Other sections were processed for immunohistochemistry. To unmask antigen-binding sites, a citrate buffer (0.1 M, pH 6.0) treatment for 40 min at 94 °C was used. After cooling on ice, endogenous peroxidases (methanol 10%, H_2_O_2_ 3% in PBS) were blocked, permeabilized with a GS-PBS-T solution (goat serum 1%, Triton X-100 0.4%), and then blocked with GS 3.5% -PBS-T for 45 min. Samples were incubated overnight with the primary antibody, mouse anti-insulin (1:1,000 ab181547 from Abcam, Cambridge, UK) prepared in 3.5% GS-PBS-T. A biotinylated secondary antibody [goat anti-mouse immunoglobulin G (IgG), Vector, BA-9200] was used. The signal was amplified with ABC (Vector, Burlingame, USA), for its subsequent reaction with 3,3’-diaminobenzidine (DAB) (Sigma-Aldrich, San Luis, USA). Samples were counterstained with cresyl violet (Sigma-Aldrich, San Luis, USA), dehydrated and mounted with Eukit (PanReac AppliChem, Castellar del Vallès, Spain).

Five separate fields from pancreatic sections were assessed at 10× and 20× objectives, and images were captured using a light microscope (Nikon Eclipse E800, Tokyo, Japan) and analyzed using ImageJ (https://imagej.nih.gov/ij/index.html).

Pancreatic injury was assessed in HE-stained tissue sections using a semi-quantitative scoring system (0-3) based on inflammatory cell infiltration and edema: 0 = absent; 1 = mild; 2 = moderate; 3 = severe.

### Hydrogen peroxide determination

Hydrogen peroxide (H_2_O_2_) levels in pancreatic tissue were measured using the Amplex Red Hydrogen Peroxide/Peroxidase Assay Kit (Thermo Fisher Scientific, Rockford, IL, USA). Briefly, samples were homogenized in PBS, and H_2_O_2_ concentrations were measured according to the manufacturer’s instructions. The reaction product was quantified by measuring absorbance at 560 nm. H_2_O_2_ levels were normalized to the total protein concentration of each sample.

### Statistical analysis

Data are presented as mean ± standard deviation (SD). Statistical comparisons of quantitative variables were performed using one-way analysis of variance (ANOVA) followed by Tukey’s post-hoc test. Differences were considered statistically significant at *P* < 0.05. For label-free proteomic analyses, proteins were identified with > 95% confidence and a false discovery rate (FDR) below 1%. Differential expression between groups was evaluated using Welch’s *t*-test. Significance was considered at *P* < 0.05. The results were analyzed using GraphPad Prism 8 (GraphPad Software, Inc., San Diego, CA, USA).

## RESULTS

### *Pg*NVs reduce the transcriptional expression of proinflammatory cytokines in RAW 264.7 after incubation with LPS

**Figure 2:**
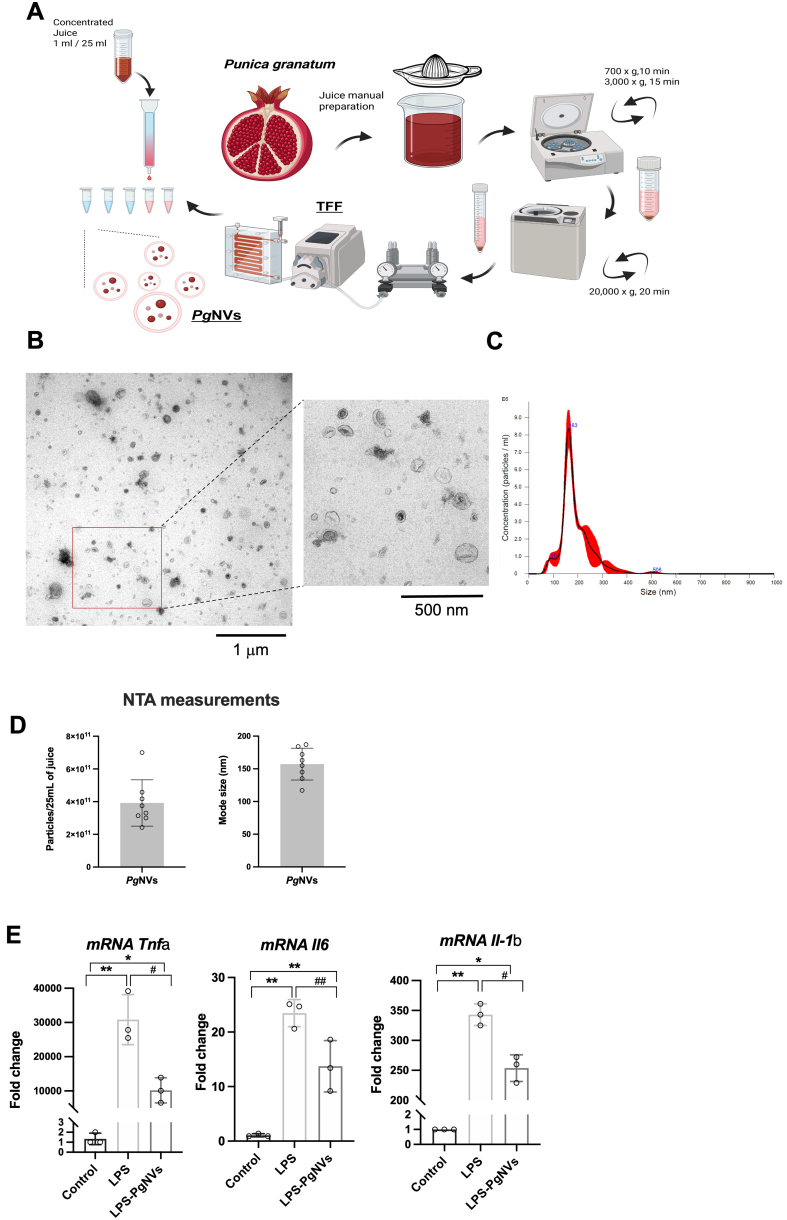
Characterization of *Pg*NVs and their inhibitory effects on LPS-induced proinflammatory cytokine expression in RAW 264.7 macrophages. (A) Schematic representation of *Pg*NVs isolation using differential centrifugation, TFF, and SEC; (B) Representative TEM obtained from *Pg*NVs; (C) Representative NTA images obtained from *Pg*NVs; (D) Determinations of particles/protein ratio and mode size by NTA and protein quantification; (E) Relative mRNA expression of *Tnfa, Il6 and Il1b* in RAW 264.7 cells incubated with LPS or LPS and *Pg*NVs (*n* = 3). *Tbp* was used as a housekeeping gene. Measurements expressed as mean ± SD. *Significantly different from control group, *P* < 0.05; **significantly different from control group, *P* < 0.01; ^#^significantly different from LPS group, *P* < 0.05; ^##^significantly different from LPS group *P* < 0.01. Created in BioRender. MARCILLA, A. (2026). https://BioRender.com/pi0m2pq. EVs: Extracellular vesicles; LPS: lipopolysaccharide; RAW: murine macrophage cell line RAW 264.7; *Pg*NVs: pomegranate-derived nanovesicles; TFF: tangential flow filtration; SEC: size exclusion chromatography; TEM: transmission electron microscopy; NTA: nanoparticle tracking analysis; mRNA: messenger RNA; *Tnfa*: *tumor necrosis factor alpha*; *Il6*: *interleukin 6*; *Il1b*: *interleukin 1 beta*; *Tbp*: *TATA-box binding protein*; SD: standard deviation.

To evaluate the effect of *Pg*NVs on RAW 264.7 murine macrophages, we first isolated *Pg*NVs as described previously, using differential centrifugation, TFF and size exclusion chromatography (SEC)^[6]^ [Figure 2A]. NTA analyses showed a mean concentration of 3.2 ± 1.23 × 10^11^ particles per 25 mL of initial processed juice, and a size mode of 157.1 ± 38.12 nm [Figure 2B and C]. TEM images also revealed various NV sizes ranging from 40 to 200 nm, in agreement with NTA [Figure 2B and D]. As expected, incubation with LPS at a concentration of 300 ng/mL for 24 h increased the transcriptional expression of proinflammatory cytokines dependent on NF-κB activity, such as *Tnfa* (*Tumor necrosis factor alpha*), *Il6* (*Interleukin 6*) and *Il1b* (*Interleukin 1 beta*). Pre-treatment with *Pg*NVs at 10 μg/mL 1 h before LPS exposure significantly reduced the expression of these proinflammatory cytokines [Figure 2E]. These results confirm the anti-inflammatory properties of *Pg*NVs in murine macrophage models.

### *Pg*NVs treatment ameliorates AP

Once the anti-inflammatory properties of *Pg*NVs were confirmed *in vitro*, we next aimed to study these effects using an experimental model of acute inflammation.

**Figure 3:**
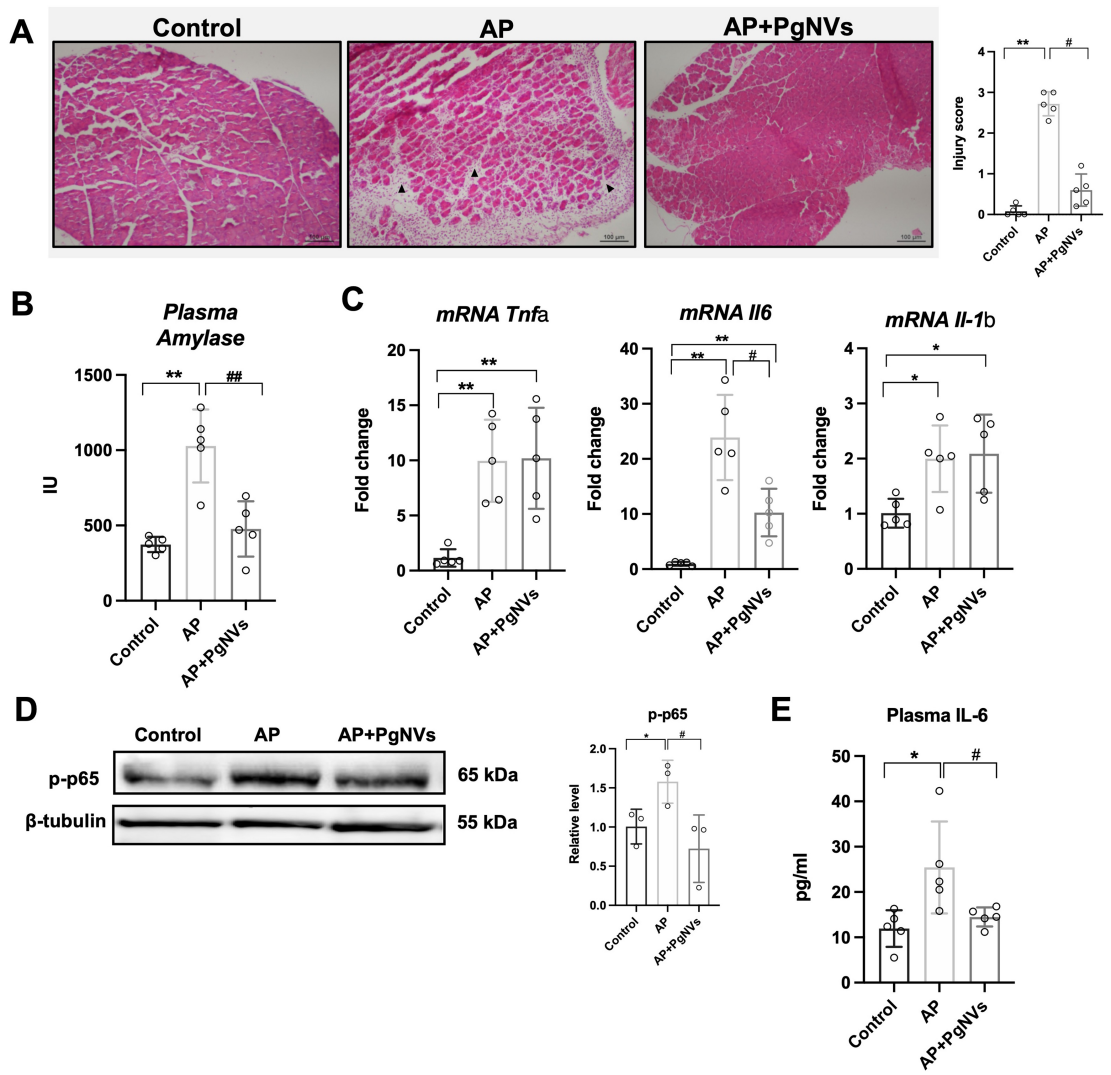
P*g*NVs administration ameliorates acute pancreatitis *in vivo*. (A) Representative images showing the mouse pancreas from each experimental group and injury score: control group showing preserved pancreatic architecture, AP group displaying marked tissue injury characterized by loss of acinar structure and inflammatory cell infiltration (black arrows), and *Pg*NVs group demonstrating partial preservation of pancreatic architecture. Samples were taken at 3 days post pancreatitis, and stained with hematoxylin-eosin staining (*n* = 5). Scale bar: 100 µm; (B) Determination of plasma amylase activity in control, AP, and AP+*Pg*NVs groups at 3 days post pancreatitis (*n* = 5); (C) Relative mRNA expression of *Tnfa, Il6 and Il1b* in the pancreas of mice three days after induction of acute pancreatitis (AP) in control, AP and AP + *Pg*NVs groups. The levels were determined by RT-qPCR analysis, using *Tbp* as housekeeping gene (*n* = 5); (D) Representative western blot image of p-p65 and densitometry using beta-tubulin as a loading control (*n* = 3); (E) Plasma IL6 levels in control, AP, and AP+*Pg*NVs groups at 3 days post pancreatitis (*n* = 5). Measurements expressed as mean ± SD. *Significantly different from control group, *P* < 0.05; **significantly different from control group, *P* < 0.01; ^#^significantly different from AP group, *P* < 0.05; ^##^significantly different from AP group, *P* < 0.01. AP: Acute pancreatitis; EVs: extracellular vesicles; *Pg*NVs: pomegranate-derived nanovesicles; mRNA: messenger RNA; *Tnfa*: *tumor necrosis factor alpha*; *Il6*: *interleukin 6* (IL6); *Il1b*: *interleukin 1 beta*; RT-qPCR: reverse transcription-quantitative polymerase chain reaction; *Tbp*: *TATA-box binding protein*; p-p65: phosphorylated p65; SD: standard deviation.

Histological analysis of pancreatic tissue in mice with pancreatitis at day 3 revealed marked differences among the experimental groups [Figure 3A]. In the control group, the pancreas displayed a normal architecture, with intact acinar cells and absence of inflammatory infiltrates and edema, resulting in an injury score of 0.08. In contrast, the AP group exhibited extensive tissue injury, characterized by loss of acinar structure, abundant inflammatory cell infiltration, and edema, as indicated by the arrows in Figure 3A. Accordingly, these alterations were reflected in a significantly increased pancreatic injury score (2.72). Finally, in the AP + *Pg*NVs group, the pancreatic parenchyma showed a more preserved histoarchitecture, with reduced inflammatory infiltration and edema compared to the untreated pancreatitis group. This histological improvement was quantitatively supported by a significantly lower injury score (0.6) [Figure 3A].

Furthermore, plasma amylase activity was higher in AP, recovering baseline values with treatment with *Pg*NVs [Figure 3B]. These results evidenced the protective properties of *Pg*NVs during the course of experimental AP.

### *Pg*NVs administration reduces Il6 up-regulation and NF-κB activation during AP

AP triggered a significant induction of the transcriptional expression of *Tnfa*, *Il6* and *Il1b* in pancreatic tissue [Figure 3C]*.* Interestingly, treatment with *Pg*NVs significantly and selectively reduced the upregulation of *Il6* during AP, without altering the expression of the other two cytokines [Figure 3C].

In addition, western blot analysis showed changes in the phosphorylation levels of the p65 subunit of transcription factor NF-κB [Figure 3D], a master regulator of the inflammatory response^[22]^, at the Ser536 site in the transactivation domain, during AP. Given the specific downregulation of *Il6* in pancreatic tissue from AP mice pretreated with *Pg*NVs, we next investigated whether this effect extended to plasma, where IL-6 is recognized as a severity marker in AP^[23]^. Indeed, AP induction produced a significant increase of IL-6 levels in plasma, which was effectively prevented by *Pg*NVs treatment [Figure 3E].

In summary, our findings confirm the anti-inflammatory properties of *Pg*NVs in an experimental model of acute inflammation, demonstrating their selective regulatory effect on *Il6* expression, consistent with our *in vitro* observations.

### *Pg*NVs treatment enhances NQO1 levels and exhibits antioxidant properties in AP

**Figure 4:**
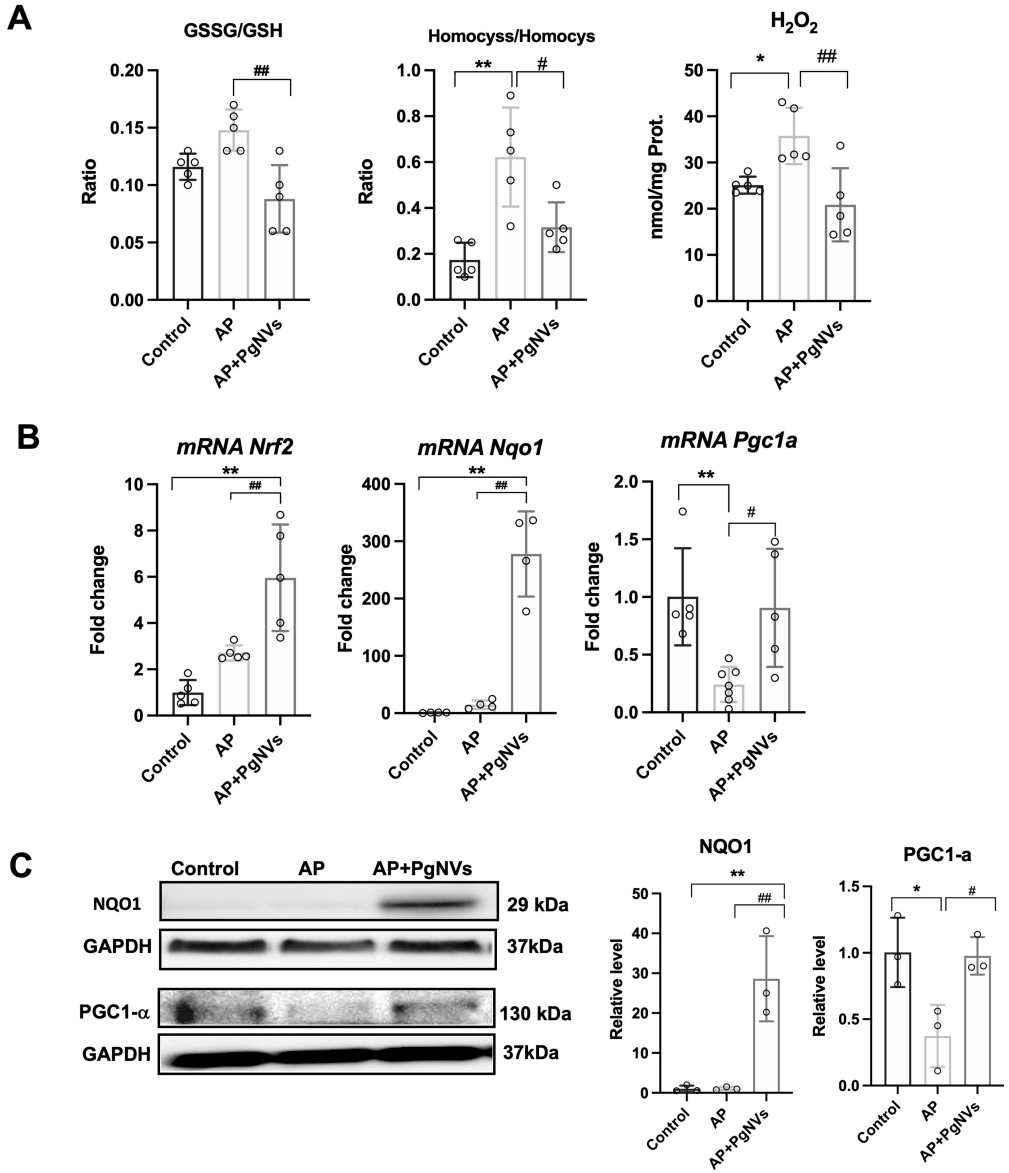
P*g*NVs treatment enhances antioxidant defense in acute pancreatitis. (A) Ratio of GSSG/GSH, ratio of Homocystine/Homocysteine, and hydrogen peroxide levels in pancreas of mice after 3 days in control, AP, and AP + *Pg*NVs groups (*n* = 5); (B) Relative mRNA levels of *Nrf2, Nqo1* and *Pgc-1α* in pancreas from mice after 3-days of pancreatitis in control, AP, and AP + *Pg*NVs groups. Expression was determined by RT-qPCR analysis, using *Tbp* as housekeeping gene (*n* = 5); (C) Representative western blot of NQO1 and PGC-1α and densitometry, using GAPDH as a loading control (*n* = 3). Measurements expressed as mean ± SD. *Significantly different from control group, *P* < 0.05; **significantly different from control group, *P* < 0.01; ^#^significantly different from AP group, *P* < 0.05; ^##^significantly different from AP group, *P* < 0.01. AP: Acute pancreatitis; *Pg*NVs: pomegranate-derived nanovesicles; GSSG: oxidized glutathione; GSH: reduced glutathione; mRNA: messenger RNA; *Nrf2*: *nuclear factor erythroid 2-related factor 2*; *Nqo1*: *NAD(P)H quinone dehydrogenase 1* (NQO1); *Pgc-1α*: *peroxisome proliferator-activated receptor gamma coactivator 1 alpha* (PGC-1α); RT-qPCR: reverse transcription-quantitative polymerase chain reaction; *Tbp*: *TATA-box binding protein*; GAPDH: glyceraldehyde-3-phosphate dehydrogenase; SD: standard deviation.

We evaluated GSSG/GSH and HCyss/HCys ratios, both well-established markers of OS and redox imbalance^[24,25]^. As shown in Figure 4A, arginine-induced AP increased both ratios. Notably, treatment with *Pg*NVs effectively counteracted this oxidative imbalance, preventing the increase of these two ratios during AP [Figure 4A]. In addition, H_2_O_2_ levels were determined and were found to be increased in mice with AP, whereas *Pg*NVs treatment significantly reduced H_2_O_2_ levels in animals with pancreatitis, restoring them toward control values [Figure 4A].

Next, transcriptional expression of *Nrf2* [*nuclear factor (erythroid-derived 2)-like 2*] was analyzed. *Nrf2* expression was increased after AP induction. Notably, mice treated with *Pg*NVs showed a further upregulation in *Nrf2* expression compared to untreated pancreatitis animals [Figure 4B].

To evaluate whether NQO1, an antioxidant enzyme^[26]^, was involved in the effect of *Pg*NVs on AP, we next analyzed both its transcriptional and protein levels. Strikingly, *Pg*NVs administration led to a dramatic increase of NQO1 expression at both mRNA and protein levels, following AP induction [Figure 4B and C], suggesting that some of the beneficial effects observed with *Pg*NVs pretreatment in AP may be mediated by NQO1 upregulation.

We also evaluated the expression of PGC-1α, a transcriptional cofactor involved in mitochondrial biogenesis and antioxidant defense^[27]^. The induction of AP resulted in a downregulation of the transcriptional expression and protein levels of PGC-1α, which was effectively prevented in mice pre-treated with *Pg*NVs [Figure 4B and C].

### *Pg*NVs treatment protects mice from AP-induced glucose intolerance

**Figure 5:**
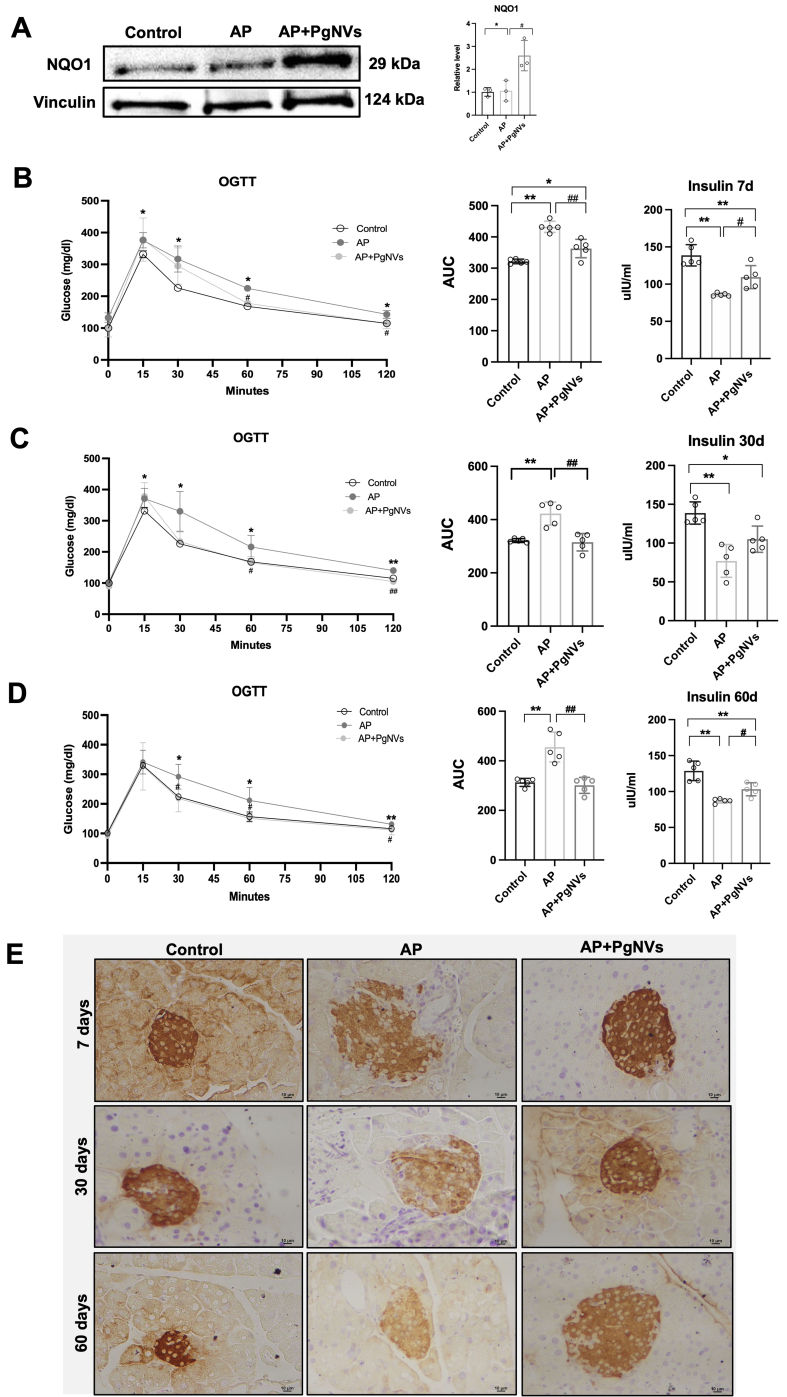
P*g*NVs treatment protects mice from AP-induced glucose intolerance. (A) Western blot of NQO1 and densitometry at 7 days after inducing AP, using vinculin as a loading control (*n* = 3); (B) OGTT indicated as the AUC, and plasma insulin levels in control, AP, and AP+*Pg*NVs groups 7 days after inducing acute pancreatitis; (C) OGTT, showing the AUC and plasma insulin levels in control, AP and AP+*Pg*NVs groups 30 days after acute pancreatitis; (D) Oral glucose test tolerance, showing the AUC and plasma insulin levels in control, AP, and AP + *Pg*NVs groups 60 days after inducing acute pancreatitis; (E) Insulin detection showing representative images taken at 10× magnifications for each experimental group: control, AP, and AP + *Pg*NVs at 7, 30 and 60 days. Scale bar: 10 µm. Measurements expressed as mean ± SD. *Significantly different from control group, *P* < 0.05; **significantly different from control group, *P* < 0.01; ^#^significantly different from AP group, *P* < 0.05; ^##^significantly different from AP, *P* < 0.01. AP: Acute pancreatitis; *Pg*NVs: pomegranate-derived nanovesicles; NQO1: NAD(P)H quinone dehydrogenase 1; OGTT: oral glucose tolerance test; AUC: area under the curve; SD: standard deviation.

Endocrine function was evaluated 7, 30 and 60 days after the induction of AP. Notably, after 7 days of AP induction, NQO1 protein levels remained elevated in AP mice treated with *Pg*NVs, suggesting a sustained protective effect [Figure 5A]. In terms of endocrine function, AP mice showed glucose intolerance compared to the control group, as evidenced by a 34% increase in the AUC during glucose tolerance testing [Figure 5B].

However, *Pg*NVs administration in AP mice improved glucose tolerance, significantly decreasing the AUC by 16% compared to the AP group [Figure 5B]. Consistently, plasma insulin levels decreased in AP mice compared to control, while *Pg*NVs treatment increased plasma insulin levels compared to the untreated group [Figure 5B].

To determine whether the endocrine insufficiency found in mice with pancreatitis persisted over longer terms and the protective effects of *Pg*NVs were maintained, we extended our analysis to 30 and 60 days after AP induction. Notably, no significant differences in the weight of the mice were observed among the experimental groups (data not shown). However, glucose tolerance tests indicated that AP mice had poorer glucose tolerance than controls at 30- and 60-days post-induction, with AUC increases of 31% and 45%, respectively [Figure 5C and D]. Remarkably, *Pg*NVs treatment in AP fully restored glucose tolerance to control levels at both time points [Figure 5C and D]. As observed at 7 days post-induction, AP mice also showed decreased plasma insulin levels at 30 and 60 days. Treatment with *Pg*NVs consistently restored plasma insulin levels toward control values [Figure 5C and D].

Immunohistochemical analysis confirmed a marked reduction of insulin-positive cells in the pancreatic islets of AP mice, reflecting a loss of beta cell mass. Strikingly, pre-treatment with *Pg*NVs prevented this beta-cell loss at 7, 30 and 60 days, resulting in insulin detection levels comparable to those of control animals [Figure 5E].

### *Pg*NVs prevent NF-κB activation induced by plasma EVs from AP mice

Given that AP induces severe systemic inflammation and that variations in IL-6 levels in plasma were observed at early time points following *Pg*NVs treatment [Figure 3E], we aimed to explore the role of plasma EVs in our model. To this end, we next isolated EVs from plasma either from mice with AP, *Pg*NVs pre-treated, or control mice (without the disease) at 3 days post pancreatitis using differential centrifugation and SEC.

**Figure 6:**
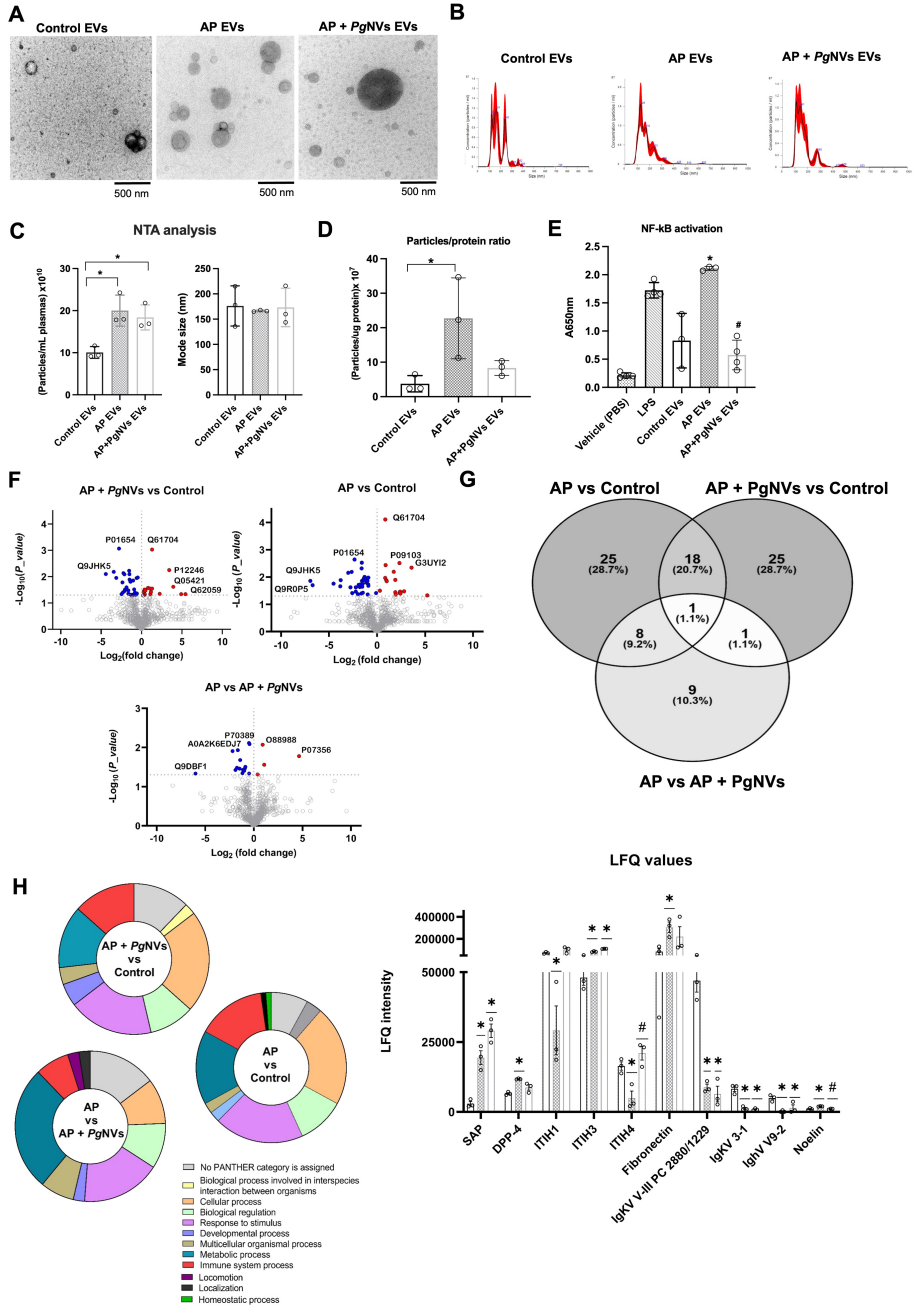
P*g*NVs induce proteomic variations on plasma EVs from AP mice and prevent NF-κB activation. (A) Representative images from TEM of plasma EVs from different mouse groups; (B) Representative images of NTA of plasma EVs from the different groups; (C) Determinations of particles/plasma ratio and mode size by NTA; (D) Particle/protein ratio, calculated as the number of particles per microgram of protein; (E) NF-κB levels in RAW 264.7 cells incubated with LPS (positive control), and EVs isolated from plasma of control, AP, and AP + *Pg*NVs mice groups at 3 days post pancreatitis; (F) Volcano plots from proteomic analysis comparing AP EVs *vs*. control EVs; AP + *Pg*NVs EVs *vs*. control EVs; and AP EVs *vs*. AP + *Pg*NVs EVs. Red and blue dots represent proteins with *P* < 0.05 and significant Log2 fold change, respectively; while gray dots indicate non-significant differences; (G) Venn diagram displaying common and condition-specific proteins with statistically significant variations detected in plasma EVs isolated from AP, AP + *Pg*NVs, and control mice groups; (H) GO classification of plasma EV proteins grouped as “biological process”, and LFQ intensity of proteins whose levels were significantly altered by AP or AP + *Pg*NVs treatments. Measurements expressed as mean ± SD. *Significantly different from control group; ^#^significantly different from AP, *P* < 0.05. AP: Acute pancreatitis; EVs: extracellular vesicles; *Pg*NVs: pomegranate-derived nanovesicles; NF-κB: nuclear factor kappa B; TEM: transmission electron microscopy; NTA: nanoparticle tracking analysis; RAW: murine macrophage cell line RAW 264.7; LPS: lipopolysaccharide; LFQ: label-free quantification; GO: Gene Ontology; SD: standard deviation.

NTA analyses revealed that mice with AP and *Pg*NVs pre-treated mice with AP produced more EVs (20.01 ± 3.68 × 10^10^ particles/mL; and 19.71 ± 5.29 × 10^10^ particles/mL, respectively) than the control group (10.06 ± 1.39 × 10^10^ particles/mL). These results were subsequently confirmed by TEM, which validated the presence and morphology of EVs in all experimental groups [Figure 6A-C]. However, the particles/protein ratio in EVs from *Pg*NVs pre-treated mice (8.31 ± 2.3 × 10^7^ particles/μg protein) was closer to that of control mice (3.75 ± 2.37 × 10^7^ particles/μg protein) when compared to plasma EVs from mice with AP (22.71 ± 11.73 × 10^7^ particles/μg protein) [Figure 6D]. Since plasma of mice with AP showed higher levels of IL-6, we next investigated whether plasma EVs could affect the inflammatory state of RAW 264.7 murine macrophages. Interestingly, EVs isolated from the plasma of AP mice increased the levels of NF-κB in RAW 264.7 murine macrophages. However, this activation was suppressed when using EVs isolated from the plasma of AP mice treated with *Pg*NVs [Figure 6E].

To further characterize these plasma EVs, we conducted a label-free quantitative proteomic analysis of these EVs from the different experimental groups (*n* = 3) [Figure 6F and Supplementary Tables 1-5]. Proteins were considered relevant if they met stringent identification criteria, including > 95% confidence and a FDR below 1%, and the corresponding LFQ values for each individual sample are reported in Supplementary Table 1. A total of 806 proteins were identified across the three experimental groups, including typical EV-associated markers such as CD9, which was present in all conditions [Supplementary Tables 1-5]. The differential expression analysis revealed 19 proteins with significantly different levels when comparing plasma EVs from AP mice and those from AP mice pretreated with *Pg*NVs [Figure 6G and Supplementary Table 2], 54 proteins with differential levels between EVs from control and AP mice [Figure 6G and Supplementary Table 3], and 50 proteins with differential levels between EVs from control and AP mice pretreated with *Pg*NVs [Figure 6G and Supplementary Table 4].

Interestingly, when comparing the sets of significantly altered proteins across all three experimental groups (AP *vs.* AP + *Pg*NVs, AP *vs.* Control, and AP + *Pg*NVs *vs.* Control), distinct overlaps were observed [Figure 6G and Supplementary Tables 2-5]. Among the 18 proteins shared between the AP *vs.* Control and AP + *Pg*NVs *vs.* Control comparisons, 10 were immunoglobulins, reflecting the loss of the physiological immunoglobulin signature in plasma EVs during AP. Three representative immunoglobulins (IgKV V-III PC 2880/1229, IgKV 3-1, and IghV V9-2) are shown in Figure 6H to illustrate this pattern. In contrast, 4 proteins within this shared group, including Serum Amyloid P-component (SAP), remained elevated in both AP and AP + *Pg*NVs, reflecting persistent inflammatory signals not fully reversed by *Pg*NVs treatment [Figure 6H and Supplementary Tables 2-5]. Other proteins displayed distinct patterns consistent with their functional roles. Fibronectin and dipeptidyl peptidase-4 (DPP4) showed elevated levels in AP plasma EVs. *Pg*NVs treatment did not induce statistically significant changes *vs.* AP; however, protein levels showed a tendency toward normalization, reaching values not significantly different from those of control EVs. In addition, a group of eight proteins shared between the AP *vs.* Control and AP *vs.* AP + *Pg*NVs comparisons exhibited a clear restoration pattern, with protein levels in the AP + *Pg*NVs group resembling those observed in control EVs. This group includes Noelin (OLFM1), inter-alpha-trypsin inhibitor heavy chains 1 and 4 (ITIH1 and ITIH4), vitronectin, complement factor H-related proteins, immunoglobulin heavy variable 1-84, and prolyl endopeptidase FAP (fibroblast activation protein) [Figure 6H and Supplementary Tables 2-5].

Inter-alpha-trypsin inhibitor heavy chain H3 (ITIH3) was the only protein found to be significantly altered across all three comparisons. Notably, ITIH3 levels were markedly elevated in both AP and AP + *Pg*NVs EVs relative to control, indicating that AP strongly increases its abundance. *Pg*NVs treatment was associated with a further increase in ITIH3 levels; however, the modest difference between AP and AP + *Pg*NVs EVs contrasts with the pronounced restoration observed for ITIH1 and ITIH4, which showed reduced levels in AP EVs compared with control and recovered toward control levels following *Pg*NVs administration [Figure 6H and Supplementary Tables 2-5].

Additionally, Gene Ontology (GO) biological process analysis revealed that while most protein classifications showed minimal variation among groups, the categories ‘Metabolic process’ and ‘Immune system process’ exhibited notable differences. Specifically, ‘Metabolic process’ accounted for 14.78%, 26.83%, and 13.41% of total process hits in the AP + *Pg*NVs vs AP, AP vs Control, and AP + *Pg*NVs vs Control comparisons, respectively, whereas ‘Immune system process’ represented 7.3%, 12.2%, and 14.7% in these same comparisons [Figure 6H].

## DISCUSSION

Numerous reports have described the anti-inflammatory and antioxidant properties of the pomegranate extracts in metabolic, cardiovascular and inflammatory diseases, including pancreatitis^[28,29]^. Traditionally, these beneficial effects have been attributed to the combination of their bioactive compounds, including punicalagins, punicalins, gallic acid, and anthocyanins^[30]^. Although many mechanisms have been described to explain these effects, our group has recently identified NVs as a major component of pomegranate juice with potential therapeutic applications^[6]^.

This study provides the first evidence of the protective role of *Pg*NVs administration on AP progression, adding *Pg*NVs to the list of studies highlighting PDNVs as promising therapeutics both *in vitro* and *in vivo* as anticancer, anti-inflammatory and antioxidant agents^[4,31,32]^.

*Pg*NVs treatment significantly reduced the inflammatory response and OS in the initial stages of the disease in an experimental model of AP in males, also improving endocrine dysfunction secondary to pancreatic damage. Only males were used in this study to ensure comparability with previous studies on experimental pancreatitis and pomegranate-derived intervention^[29]^. Moreover, estrogens have been reported to exert protective effects in experimental pancreatitis^[33]^, which could confound data interpretation if females were included.

In agreement with *in vitro* findings, we observed that a single dose of 10 µg of *Pg*NVs administered subcutaneously was effective. The rationale for this dose selection was initially guided by our own *in vitro* studies, in which *Pg*NVs at 10 µg/mL efficiently modulated macrophage responses. Although *in vitro* effects are often amplified compared to *in vivo* settings, these results provided a rational basis for selecting a total dose of 10 µg for animal experiments. Moreover, this dose is in line with those commonly used in NV-based therapeutic studies^[34]^, despite the wide variability reported in the literature, where administered EV doses range from 0.1 µg to several hundred micrograms or even milligrams of vesicle protein, and particle numbers vary between 10^6^ and 10^11^^[34]^. Accordingly, the experimental dose used in this study (~5 × 10^10^ particles) falls within this established range. Nevertheless, further research will be needed to determine whether repeated administrations or higher doses of *Pg*NVs could yield stronger therapeutic effects. Furthermore, our previous studies evidenced no cytotoxic effects of *Pg*NVs in several human cell lines, including Caco-2 (intestinal epithelial) and THP-1 (macrophage-like) cells^[6]^.

*Pg*NVs in AP reduced phosphorylation of the p65 subunit, responsible for the transcriptional activity of NF-κB^[22]^. However, when we analyzed the expression of proinflammatory cytokines, *Pg*NVs administration selectively downregulated the transcriptional levels of *Il6* after an AP episode. Additionally, *Pg*NVs prevented the increase in IL-6 observed in pancreatitis, which is considered a marker of severity in AP and is responsible for systemic damage and inflammation^[35]^.

In a previous work, we showed a specific mechanism of selective repression of *Il6* through blocking p65 phosphorylation. We demonstrated that PGC-1α binds to p65 and prevents its activation and the subsequent upregulation of *Il6*^[27]^. PGC-1α is a transcriptional co-activator that fundamentally regulates mitochondrial biogenesis and oxidative metabolism, but also exhibits anti-inflammatory properties^[36]^. In fact, previously we confirmed that PGC-1α deficiency exacerbates pancreatic damage in obese mice with AP^[27]^. In this study, we observed that AP reduces PGC-1α levels, while the administration of *Pg*NVs prevents this downregulation. This suggests that the selective decrease in *Il6* upregulation may be partly due to the maintenance of PGC-1α levels. Similarly, previous studies showed that pomegranate extract increased PGC-1α levels, exerting antioxidant and antihypertensive properties in an experimental rat model^[37]^. Interestingly, a recent report has shown that *Pg*NVs isolated by differential centrifugation displayed powerful antioxidant effects on mice pancreatic acinar cells^[38]^. Surprisingly, these *Pg*NVs did not induce significant variations in IL-6 secretion in acinar cells pre-treated with plasma EVs isolated from mice with taurocholic acid-induced AP^[38]^. Of note, these authors used *Pg*NVs isolated by differential centrifugation, whereas we have used combined differential centrifugation, TFF and SEC, which renders pure *Pg*NVs preparations lacking most of nucleic acids and protein aggregates co-isolated by other techniques^[39]^. The isolation method, along with the different models of study employed, can greatly influence the observed effects^[4,40]^.

As previously mentioned, OS plays a crucial role in amplifying the inflammatory response in AP. Experimental evidence indicates that prophylactic treatment with antioxidants can reduce pancreatic damage in experimental models of AP^[41]^. In previous investigations, we observed that AP induced by taurocholate in rats produced an increase in the GSSG/GSH and HCyss/HCys ratios, indicative of OS^[42]^. Here, using the experimental model of pancreatitis induced by arginine, we obtained a similar oxidation profile. Notably, the administration of *Pg*NVs prevented oxidation and maintained levels comparable to those in control mice, confirming the antioxidant properties shown *in vitro*. These findings suggest that the protective role of *Pg*NVs in modulating the inflammatory response in AP might be partly due to their antioxidant capacity.

Plant extracts are known to regulate the expression of different antioxidant enzymes such as NQO1^[43]^. NQO1 is an enzyme of the quinone family with multiple functions, notably providing antioxidant and anti-inflammatory benefits^[26]^. Recent studies have demonstrated that pomegranate juice extract increases the expression of NQO1 in the intestinal epithelial cell line IEC-6, reducing intracellular reactive oxygen species (ROS) levels^[44]^. Our findings show a dramatic increase in NQO1 expression, both at the transcriptional and protein levels, in mice with AP that have been treated with *Pg*NVs. Shen *et al*. demonstrated that pharmacological stimulation of NQO1 with dunnione protected mice against cerulein-induced AP in a ROS-dependent mechanism^[45]^. Additionally, and related to our results showing selective modulation of *Il6* expression with *Pg*NVs treatment, NQO1 has been shown to selectively suppress Toll-like receptor (TLR) ligand-induced IL-6 in macrophages^[46]^. Furthermore, NQO1 binds and protects PGC-1α from degradation and may also contribute to the *Il6* repression mechanism discussed above^[47]^. Considering the discussed results, we propose that the increase in NQO1 after the administration of *Pg*NVs in mice with pancreatitis may contribute to reducing the inflammatory response both directly and by decreasing OS. In addition, transcriptional analysis also revealed that *Nrf2* expression was upregulated following pancreatitis induction and further enhanced in *Pg*NVs-treated animals, suggesting that the increase in NQO1 may, at least in part, be mediated by Nrf2. Previous studies have reported similar *Nrf2* upregulation in experimental models of pancreatitis, including cerulein-induced pancreatitis, where Nrf2 ameliorates defective autophagic processes and inhibits ferroptosis through suppression of Beclin1-Slc7a11 complex formation^[48,49]^. Future studies are needed to clarify the mechanisms by which *Pg*NVs increase Nrf2-NQO1 levels, including the potential contribution of the Nrf2 pathway to this effect.

In recent years, the role of EVs has been studied in the pathogenesis of inflammatory diseases^[50]^. In the context of AP, EVs have been found to play a critical role in systemic complications, specifically in the activation of alveolar macrophages responsible for lung damage^[51,52]^. In our study, plasma EVs isolated from mice with AP exhibited a markedly higher particle-to-protein ratio compared with control animals. Notably, this ratio was partially normalized in mice with pancreatitis treated with *Pg*NVs, reaching values closer to those observed in the control group. Although the particle-to-protein ratio is commonly used as an indicator of EV purity^[53]^, when isolation protocols are kept constant, variations in this parameter may also reflect disease-associated changes in EV abundance and cargo composition^[54]^, particularly under inflammatory conditions such as AP.

In line with this notion, plasma EVs derived from mice with pancreatitis induce the activation of NF-κB in RAW 264.7 murine macrophages. In contrast, plasma EVs isolated from mice with pancreatitis and treated with *Pg*NVs produced a response similar to that of control mice. Proteomic analyses identified a shift in EV composition reflected in the enrichment of inflammation-associated proteins in AP-derived EVs. Hence, fibronectin, a key extracellular matrix protein linked to NF-κB activation^[55]^, was elevated in EVs from AP mice, while its levels in the AP + *Pg*NVs group were reduced to values similar to those of controls, consistent with the observed suppression of NF-κB activation in RAW 264.7. Additionally, FAP was enriched in AP-EVs, suggesting active stromal remodeling^[56]^. Furthermore, we observed an upregulation of DPP4 in EVs of mice with induced AP. DPP4 is a serine protease that promotes the inflammatory response in multiple tissues through NF-κB activation^[57,58]^, and interestingly, it also appears elevated in the plasma of patients with type 2 diabetes and pancreatic ductal adenocarcinoma^[59,60]^. *Pg*NVs administration reduced DPP4 levels in EVs following AP to values similar to those of controls. On the other hand, a decrease in the levels of inter-alpha-trypsin inhibitor protein heavy chain 4 (ITIH4) was observed in EVs isolated from mice with induced AP. ITIH4 is a protein with known anti-inflammatory potential often used as a marker of acute ischemic stroke and rheumatoid arthritis due to its downregulation in these conditions^[61,62]^. Other members of the inter-alpha-trypsin inhibitor family, including ITIH1 and ITIH3, have also been implicated in both pro- and anti-inflammatory processes and extracellular matrix regulation^[63]^. *Pg*NVs administration in the model of pancreatitis restored the levels of ITIH4 and ITIH1 toward control levels, while ITIH3 remained highly elevated in AP EVs and increased slightly further after *Pg*NVs treatment, reflecting the context-dependent roles of these proteins in inflammation. *Pg*NVs administration in the model of pancreatitis restored the levels of this anti-inflammatory protein in the plasma EVs of these mice. In contrast, cytochrome P450 2E1 (CYP2E1), a key enzyme of the cytochrome P450 family, was specifically upregulated in EVs from AP + *Pg*NVs mice. Interestingly, this pleiotropic enzyme is linked to ROS generation^[64]^. The release of CYP2E1 through EVs from the tissue could contribute to the enhanced antioxidant response triggered by *Pg*NVs administration and observed through NQO1 dramatic upregulation in the pancreatic gland. Notably, EVs from control plasma exhibited a significant presence of immunoglobulins, whereas this signature was lost in AP. Under physiological conditions, circulating EVs might reflect the normal immune equilibrium, carrying immunoglobulins as part of their surface corona^[65]^. However, during AP, tissue-derived EVs may enter circulation, masking this immunoglobulin signature. The differences observed in the levels of these proteins, along with variations in other molecules within these EVs (such as RNA, DNA, and others), may explain the variations in the inflammatory potential of plasma EVs. Future research is needed to understand how these differences impact systemic inflammation and the overall health of the patient.

Endocrine dysfunction secondary to AP is one of the complications that has generated the most interest at a clinical level in recent years^[12,66]^. Due to the lack of experimental models developed for the study of this complication, the underlying mechanisms are unknown, although the loss of beta cells is suggested as the main etiopathogenic factor^[11]^. In this study, we established an experimental model of type 3 diabetes by inducing AP with arginine in mice. Our results show a worse endocrine function from 7 days after the induction of pancreatitis that is maintained at 30 and 60 days. We observed a loss of beta cells that results in a lower release of insulin in plasma and subsequent intolerance to oral glucose overload. Strikingly, *Pg*NVs administration improved endocrine function 7 days post-induction, with nearly complete recovery observed at 30 and 60 days. However, although *Pg*NVs restored glucose tolerance at 60 days post-AP, plasma insulin levels showed only partial recovery. This apparent discrepancy may be explained by compensatory mechanisms that maintain glucose homeostasis without full normalization of insulin levels, together with a time-dependent effect of the single *Pg*NVs dose, which appears more effective at earlier stages. Importantly, OGTT reflects an integrated physiological response, whereas insulin measurements are static, and thus do not necessarily correlate. In line with this temporal pattern, 7 days post-induction, mice with induced pancreatitis and treated with *Pg*NVs still showed high levels of NQO1. Previous studies have demonstrated that supplementation with NAD^+^, a cofactor produced through NQO1 activation^[67]^, restored insulin secretion in both murine and human β-cells when affected by inflammatory cytokines^[68,69]^. Furthermore, NQO1-deficient mice showed increased mortality in pancreatic beta cells in a streptozotocin-induced diabetes model^[70]^, suggesting that the maintenance of beta cell integrity after *Pg*NVs administration in AP may be attributed to the modulation of NQO1 levels.

Our findings are supported by studies exploring the beneficial effects of pomegranate total extract on DM through its anti-inflammatory and antioxidant properties. Pomegranate juice supplementation was able to improve the structure of the pancreas in a streptozotocin-nicotinamide-induced type 2 diabetic Sprague-Dawley rats experimental model^[71]^. In another study, oral administration of pomegranate seed juice reduced the levels of lipid peroxidation biomarkers such as malondialdehyde and increased total antioxidant status in diabetic rats^[72]^. In the context of diabetes secondary to chronic pancreatitis, and using an LPS-induced experimental mouse model, pomegranate extract prevented exocrine and endocrine insufficiencies by preventing the nuclear translocation of NF-κB^[28]^. The beneficial effects observed in animal models have been transferred to clinical practice. Indeed, it has even been observed that consuming pomegranate juice can serve as a complementary treatment in patients with hyperglycemia and especially as a prophylactic agent due to its ability to increase peripheral insulin levels^[73]^. Given that *Pg*NVs might be an important constituent of the juice, we can hypothesize that some of the beneficial effects observed in these studies might be a consequence of *Pg*NVs action, in collaboration with other components of the juice (such as phenolic compounds). Our group identified the proteomic content of *Pg*NVs in previous studies^[6]^. Also, other authors described their microRNA content^[74]^. Future studies might contribute to identifying other components within *Pg*NVs involved in its therapeutic properties with potential future clinical applications.

This study was performed exclusively in male mice; we acknowledge that this represents a limitation, as sex-specific differences in disease progression and response to *Pg*NVs may exist, and future studies including female animals should address this gap. Additionally, the current work did not employ transgenic or knockout models, which would allow a more precise dissection of the molecular mechanisms underlying the protective effects of *Pg*NVs. These aspects should be considered when interpreting the results and will be addressed in future investigations.

In summary, the present study shows for the first time the protective role of *Pg*NVs in experimental AP by demonstrating their anti-inflammatory and antioxidant properties. Administration of *Pg*NVs in AP reduced the inflammatory response by decreasing NF-κB activation and selectively repressing *Il6* expression. In addition, OS levels diminished through the upregulation of NQO1 levels. As a consequence, endocrine dysfunction secondary to AP was avoided due to the maintenance of the integrity of pancreatic beta cells.

## DECLARATIONS

### Availability of data and materials

Some results supporting the study are presented in the Supplementary Materials. Other raw data that support the findings of this study are available from the corresponding author upon reasonable request.

### Copyright

© The Author(s) 2026.
